# Static graph approximations of dynamic contact networks for epidemic forecasting

**DOI:** 10.1038/s41598-024-62271-0

**Published:** 2024-05-22

**Authors:** Razieh Shirzadkhani, Shenyang Huang, Abby Leung, Reihaneh Rabbany

**Affiliations:** 1 Mila, Quebec Artificial Intelligence Institute, Montreal, Canada; 2 School of Computer Science, McGill University, Montreal, Canada; 3 CIFAR AI Chair, Montreal, Canada; 4 Department of Bioresource Engineering, McGill University, Montreal, Canada

**Keywords:** Epidemiology, Computer science

## Abstract

Epidemic modeling is essential in understanding the spread of infectious diseases like COVID-19 and devising effective intervention strategies to control them. Recently, network-based disease models have integrated traditional compartment-based modeling with real-world contact graphs and shown promising results. However, in an ongoing epidemic, future contact network patterns are not observed yet. To address this, we use aggregated static networks to approximate future contacts for disease modeling. The standard method in the literature concatenates all edges from a dynamic graph into one collapsed graph, called the *full static* graph. However, the full static graph often leads to severe overestimation of key epidemic characteristics. Therefore, we propose two novel static network approximation methods, *DegMST* and *EdgeMST*, designed to preserve the sparsity of real world contact network while remaining connected. DegMST and EdgeMST use the frequency of temporal edges and the node degrees respectively to preserve sparsity. Our analysis show that our models more closely resemble the network characteristics of the dynamic graph compared to the full static ones. Moreover, our analysis on seven real-world contact networks suggests EdgeMST yield more accurate estimations of disease dynamics for epidemic forecasting when compared to the standard *full static* method.

## Introduction

Epidemic modeling of infectious diseases equips governments and public health officials with the ability to predict outbreaks and minimize associated risks through timely intervention. Recently, the newly emerged infectious disease, COVID-19, has significantly impacted multiple aspects of daily life, leading to unexpected social and economic challenges. Traditional epidemic models, known as compartment-based models, rest on the assumption of homogeneous mixing amongst individuals. Due to their simplicity, these models have been widely employed for a considerable period. Nevertheless, infectious diseases, such as COVID-19, propagate via close contacts between individuals, thereby rarely adhering to the homogeneous mixing assumption inherent to compartment-based models. Evidence suggests that compartment-based models frequently overestimate the number of infections, and as such, epidemic modeling based on human contact networks presents a more realistic approach^1,2^. It is thus vital to comprehend the structure of contact networks and integrate it into epidemic models.

One significant challenge in understanding human contact networks is their evolving nature. Connections, which are transient and subject to change, often appear or disappear at different times. Temporal graphs are highly adept at modeling these fluctuations over time and have rapidly become the preferred data representation for human contact networks. Recent works incorporating large-scale dynamic graphs with traditional compartment-based epidemic models have shown promising results for forecasting COVID-19 infection trajectories^3–5^. However, when forecasting the trajectory of an ongoing epidemic (or simply epidemic forecasting), the dynamic contact network structure is inaccessible, as the future contacts have not been observed. In addition, accurately predicting future contact patterns proves challenging since the graph structures often undergo significant changes over time^6,7^. As such, utilizing a static, approximated network based on the past contact networks emerges as a more practical approach for real-time epidemic forecasting. Furthermore, due to privacy concerns, access to dynamic contact networks is often unfeasible, given that it captures large-scale, fine-grain mobility data recorded over a city or a country^8^. A practical compromise is to employ an aggregated network, which effectively maintains individual privacy while encapsulating key temporal patterns. Such an aggregated network reflects the average contact patterns over a given period, rather than detailing the precise contacts for each individual on a fine-grained scale.

Lastly, the understanding and interpretation of temporal graph structures remains an open research question and an active area of study ^9,10^. In contrast, there exists a robust body of tools and literature for the exploration of static network structure^11^. Therefore, by transforming an evolving network into a static one, we can leverage tools from the static graph literature to understand the link between network structure and disease dynamics. For example, centrality measures^12^, community mining^13^ and graph motif mining^14^ can be applied on static graphs to understand the structural roles of nodes and their effect on the spread of infectious diseases^15^. However, there is few work studying the conversion process from a dynamic network into a static one for the purpose of disease modeling.

**Figure 1:**
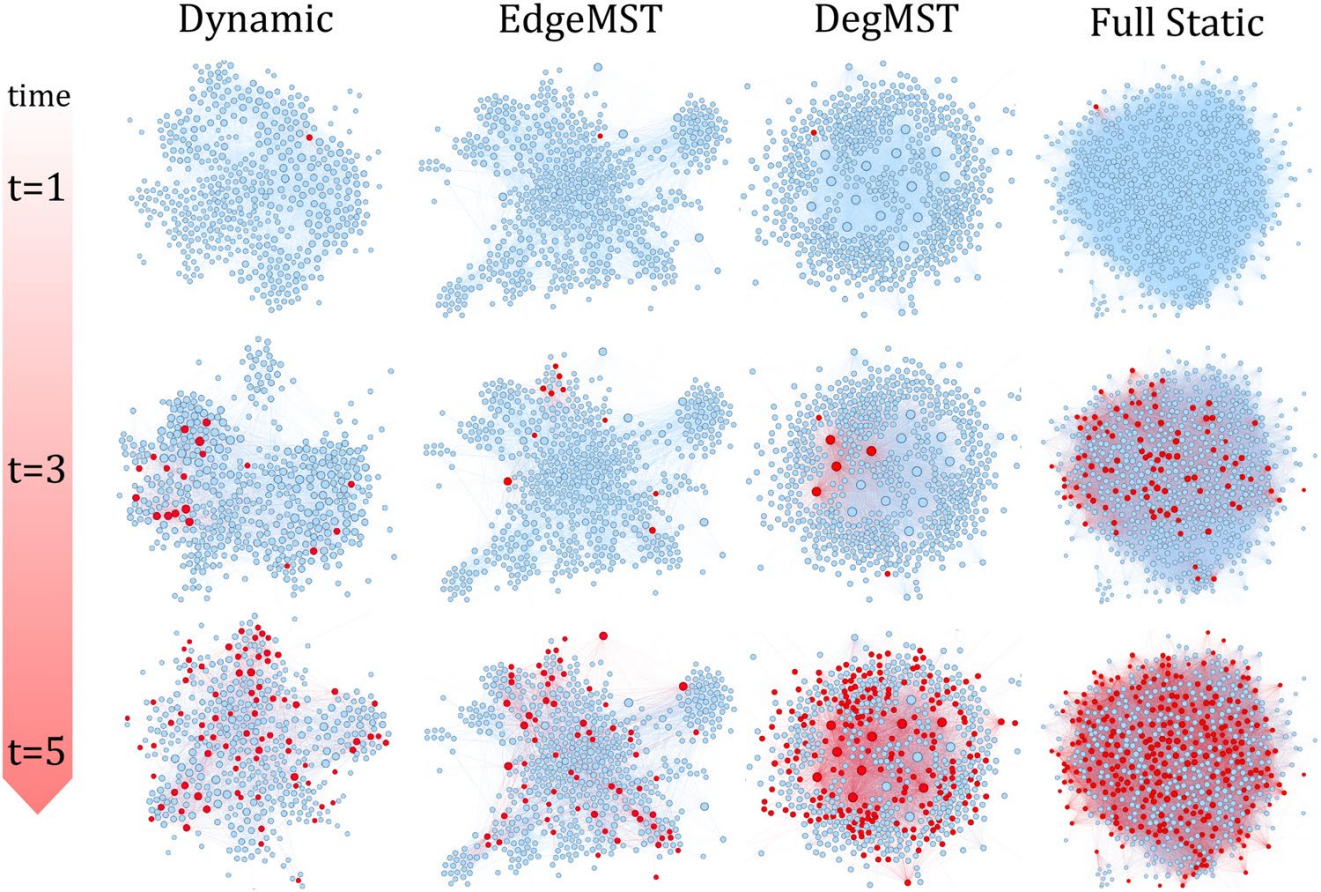
Comparison of disease spreading dynamics on the Copenhagen dataset, and its corresponding EdgeMST (ours), DegMST (ours) and full static graphs time-aggregated approximations. During these first five days, full static network severely overestimates the spread of the disease when compared to dynamic graph while our proposed EdgeMST and DegMST networks closely resemble the dynamic graph. The red nodes indicate the infected individuals.

In this work, we propose two novel methods for approximating static networks from dynamic ones, which can then be utilized for epidemic modeling and forecasting. The conventional approach for converting a dynamic graph into a static one involves collapsing all temporal edges into a single graph, a process which results in what we refer to as a *full static* graph ^9,16^. However, given the frequent addition and removal of edges in a dynamic graph, they seldom co-exist simultaneously, contrary to what is assumed in the full static graph. This leads to the overestimation of contacts and infections when a full static graph is used for disease modeling. To address this, we put forward two novel approximation algorithms for transforming a dynamic graph into a static one: the Degree Minimum Spanning Tree (*DegMST*) and the Edge Minimum Spanning Tree (*EdgeMST*) algorithms. These algorithms are designed to preserve the sparsity of the dynamic contact network while retaining its connectivity. In particular, DegMST and EdgeMST consider the node degree information and the frequency of temporal edges respectively to ensure the same level of sparsity as temporal network. In addition, DegMST and EdgeMST construct a minimum spanning tree to ensure that the network is connected.

Figure 1 illustrates the main findings of this paper, where we compare the disease spreading dynamics of the full static, DegMST, EdgeMST, and the dynamic graphs using a real-world contact network-the Copenhagen dataset-over the initial 5 days of disease spread. Infected individuals are denoted by red nodes, while the remaining individuals are marked blue. As observed, the full static graph leads to an overestimation of active infections compared to the dynamic graph. Conversely, our proposed DegMST and EdgeMST graphs more accurately represent the dynamic graph’s sparsity, structure, and number of active infections. The primary contributions of this work can be summarized as follows:

- We introduce two novel conversion methods from a dynamic graph to a static one, namely EdgeMST and DegMST, for the purpose of epidemic modeling. Both algorithms are designed to preserve the sparsity of real world contact networks while maintaining a connected network (through the use of a Minimum Spanning Tree). The frequency of temporal edges and the node degrees are taken into account in generating EdgeMST and DegMST respectively.
- We conduct experiments on seven real-world dynamic contact networks of different sizes with up to 9.5 million edges. Across all datasets, we observe that our proposed EdgeMST and DegMST significantly outperforms the standard full static approach in terms of how well they approximates the disease spread of the true contact network.
- We demonstrate that our EdgeMST algorithm is highly effective for epidemic forecasting as a proxy for future contact network. EdgeMST yields the best approximations of infection curves and other disease characteristics to that of the dynamic contact network.

## Related works

### Contact network disease modeling

Classical compartment based disease models assume homogeneous mixing between all individuals^17^. However, human contact networks are inherently heterogeneous, with contacts occurring more frequently among acquaintances. Therefore, incorporating contact networks into disease modeling facilitates more accurate reflections of real infection curves^2,18^. Static networks have been used to measure and study the effect of different epidemic values such as basic reproduction number ($R_0$)^19^, epidemic threshold^20^ and outbreak size ($\Omega$)^21^. Moreover, the structural effects of stable contact networks on epidemics have been thoroughly investigated using empirical and synthetic data^22,23^. Over the past few decades, disease modeling on dynamic networks has advanced^24,25^. It has been shown that the $R_0$ and its relation to $\Omega$ vary between dynamic and static networks and the temporal network structures affect these parameters^26^. Recently, spread of COVID-19 on dynamic networks has been used for different applications with interesting results. For example, propagation of COVID-19 in different racial and social levels of the US population was captured^4^. Also, mobility data analysis from the US showed that around 20% of individuals cause 80% of infections and only 10% of events can be considered as super-spreading events leading to massive infections^27^.

### Static network approximation from dynamic ones

However, current static network representations often fail to capture characteristics of the underlying dynamic network, limiting their capacity to accurately model disease dynamics^28^. Although various properties of static networks have been widely studied, understanding dynamic networks remains an open problem. Therefore, our objective is to construct more powerful static networks that preserve the characteristics of a dynamic contact network. The conventional method to convert a dynamic network into a static one is to aggregate all temporal edges into a collapsed static graph, often referred to as full static graph. Yet, this representation fails to serve as an ideal substitute for dynamic networks^9,16,29^. There have been prior works which suggest that an aggregated network with edges weighted for contact duration is a better estimate compared to an unweighted version^30^. However, this approach also remains imperfect, as the resultant graph is often much denser than a dynamic graph. Similar attempts have been made to incorporate dynamics into edge (weights), for instance, adding edges only when nodes have been in contact within a specified interval, or basing weights on an exponential relation to the time of contact^9,24,31^. Nevertheless, these methods often neglect the frequency of contacts and similarly result in dense graphs. A recent study explored the compression of network chronologies into a sequence of static graphs by preserving the dynamics of contacts^32^. In this study, we propose two novel methods to convert dynamic networks into a single static graph by considering both the frequency of temporal edges as well as guaranteeing a connected network with similar density to that of the dynamic graph.

## Datasets

We utilize seven human contact networks ranging from hundreds of nodes to hundreds of thousands of nodes and spanning days or even weeks. **Copenhagen** dataset was gathered from close contacts between university students using Bluetooth devices^33^. A device was attached to each student and a contact was recorded between devices that were in less than $\sim 10$m distance every 5 minute. **Conference**, **Workplace**, **Lyon school** and **High school** datasets were collected as part of the Sociopattern project using radio-frequency identification sensors (RFID) based on co-presence of individuals in specific places^34^. Each individual carried a sensor which sent a signal to the RFID readers every 20 seconds, and a contact was recorded between each two individuals which a signal was recorded from them at the same time. **Wi-Fi** dataset is extracted from connections to Wi-Fi hot-Spots in Montreal ^35^. We consider a contact between two devices that are connected to one WiFi hot-Spot at same week. We use the recorded connections from 2009/01/01 to 2010/03/07. However, since this data set evolves through time, we only monitor the individuals that have been active in the first 20 weeks and disregard the nodes that were added to the dataset between weeks 21 to 62. **SafeGraph** dataset was collected by monitoring the mobility of individuals using mobile signals, and reports the weekly number of visits from Canadian Dissemination Areas (DA) to different Point of interests (POI)^36^. We use the data recorded between 2020/05/03 and 2020/10/27 in the Montreal area. First, we extract the number of residing devices in each DA from SafeGraph *home panel* data and an Erdős-Reńyi graph is generated between residents of each DA with average degree of 10. Then, by using *visitor home cbg* data, we extract the number of visitors ($n_{i}$) from each DA to each POI. Lastly, we choose $n_{i}$ individuals from $DA_{i}$ and $n_{j}$ individuals from $DA_{j}$ population and generate a fully connected bi-partite graph between these two sets of individuals. The random graph inside each DA is constant through time, but the connections between different DAs change over time.

**Table 1:** Dataset statistics of the studied contact networks.

Data set	# Nodes	# Edges	Duration	Granularity	Dynamic deg	Static deg	Dynamic density
Copenhagen	692	188,042	28 days	5 min	20	230	0.028
Workplace	219	82,422	120 h	20 s	6	153	0.028
Lyon school	242	213,534	20 h	20 s	90	220	0.37
High school	328	350,901	50 h	20 s	43	265	0.131
Conference	403	256,253	19 h	20 s	69	365	0.171
Wi-Fi	32,005	5,836,075	62 weeks	1 s	6	239	1.83e$-4$
SafeGraph	122,757	9,583,781	29 weeks	7 days	5	23	4.24e$-5$

In all datasets edges are formed based on close proximity, i.e., the co-presence of two individuals at a location. These datasets are considered to be close proxies to real daily contact networks for infectious disease modeling^4,22,34^. More details on the datasets are presented in 1. The *Dynamic deg.* and *Static deg.* in Table 1 are the average degree of dynamic network and full static network, respectively. To calculate the Dynamic average degree, we first calculate the average degree of all the snapshots over the entire time; then, we calculate the mean of these average degrees.

## Results

The initial population in the *E*, *I* and *R* compartments in the datasets are set to be 3, 1, and 0 in small datasets, respectively. In Wi-Fi dataset these values are set to be 30, 10 and 0 and in the Safegraph are as 1200, 400 and 0, since there is a significant increase in the number of nodes of these two datasets. We set the transmission rate $\sigma = \frac{1}{5}$ and the recovery rate $\gamma =\frac{1}{14}$ based on^37^. The $\beta$ parameter is fitted to COVID-19 infection curve in Montreal, Canada and is equal to $\beta =2.7$^38^ and $\phi =0.1227$ as explained in “Supplementary Appendix”. For quantitative analysis, we use the structure and the disease curve of the dynamic network as the ground truth and measure how closely a given method approximates the ground truth. All results are reported over 50 runs.

### Epidemic forecasting on aggregated static networks

**Figure 2:**
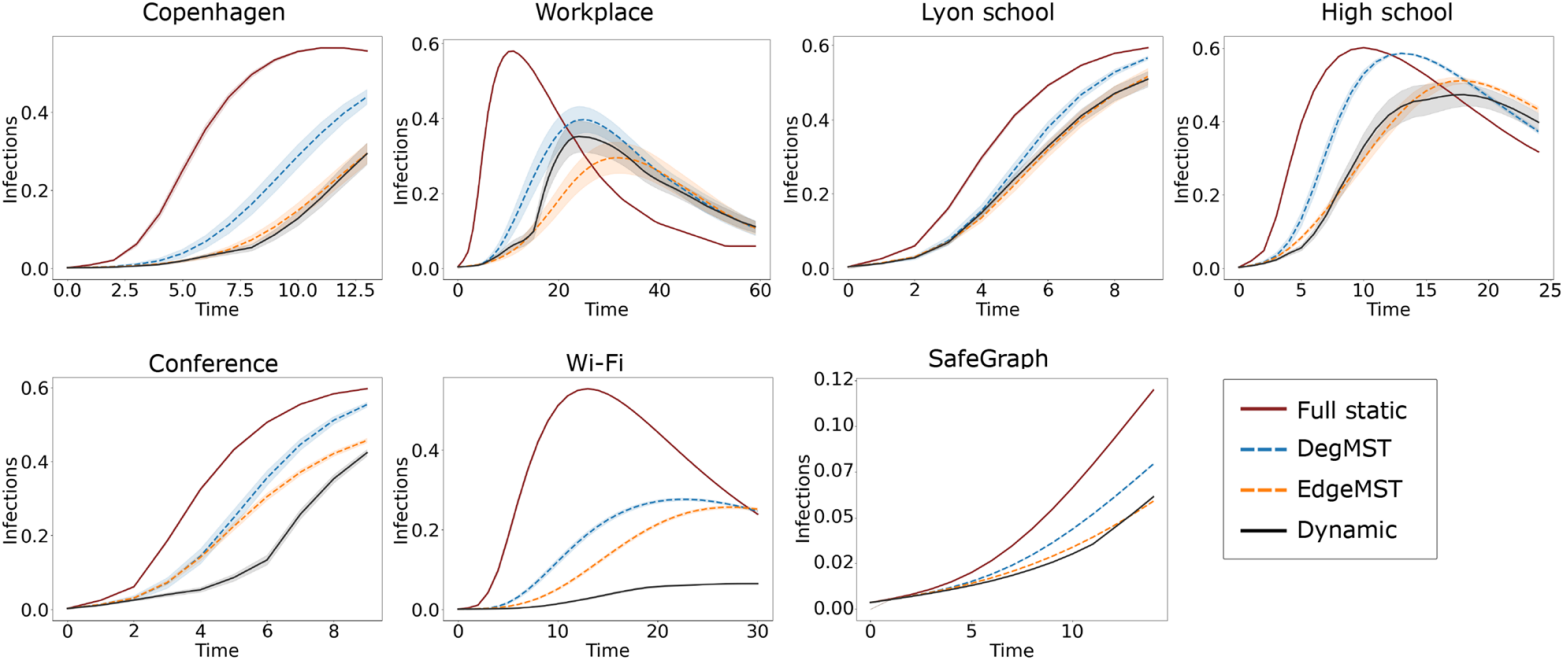
Epidemic forecasting where static networks are used as proxy for future contact networks. The networks from our EdgeMST and DegMST methods have close approximation of the disease dynamics of the true dynamic graph when compared to the full static network.

It has been shown that incorporating known (or collected) contact networks for disease modeling yields promising results^3–5^. When forecasting the active cases of an ongoing disease, the contact network structure is not observed yet. Therefore, an alternative approach is to extract information from the past contact network to construct static proxy networks for epidemic forecasting. Here we run the experiment using the first half of each dataset to generate static proxy networks, while the second half of the dynamic contact network is withheld and used to generate the ground-truth disease trajectory for testing. This assumes that the infection starts spreading through the population halfway through a dataset’s duration, we start the disease modeling at the half way point with different proxy static networks and compare the results with that from the dynamic network.

Figure 2 illustrates the fraction of active infected cases (number of active cases divided by total number of individuals) at each time step. In all datasets, both the DegMST and EdgeMST formed static networks demonstrate a closer approximation of the disease dynamics of the dynamic network when compared to the full static method. To better measure the closeness of the infection curves predictions, we use the Kullbeck-Liebler (KL) divergence^39^, $D_{KL}=(P||Q)$, to compare the distribution of the active infection curve between each static graph and the dynamic graph. This metric shows the divergence of a distribution (*P*) from a reference one (*Q*), the smaller the better. Table 2 shows KL divergence values for static graph curves seen in Fig. 2, with the dynamic network being the reference. For all datasets, the KL divergence of the full static network is the highest, meaning its active infection curve is most different from that of the dynamic graph, while DegMST and EdgeMST generated static graphs have more than three and four times smaller KL divergence, respectively.

**Table 2:**
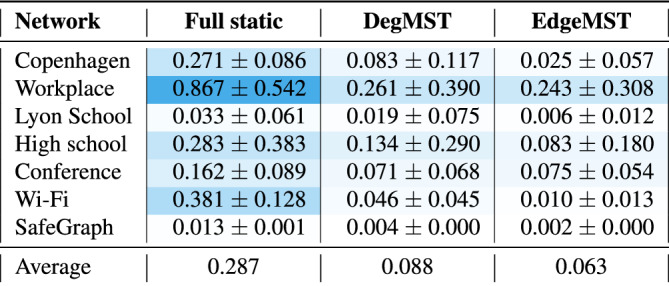
*Kullback–Leibler divergence* between various static networks and the dynamic network active infection curves presented in Fig. 2.

EdgeMST and DegMST have significantly lower KL divergence than the full static graph.

#### Observation 1

In epidemic forecasting, the active infection curves of DegMST and EdgeMST methods are three times and four times closer to that of the dynamic graph compared to the full static one based on KL divergence, respectively.

### Difference in disease characteristics

Table 3a illustrates the absolute difference between the maximum fraction of active cases obtained from the full static, DegMST, and EdgeMST approaches, compared to the values obtained from the dynamic graph results. On average, the full static graph differs from the maximum active infections by 18.5%, while the estimations from DegMST have a difference of 9.7%, and for EdgeMST, it is only 5.4%. In datasets where the infection curves have not yet reached their peak, we assume the number of infections at the last data point as the maximum infections. Table 3b, c present the absolute differences in peak time of active infections and the final attack rate of the pandemic between the static and dynamic networks, respectively. The final attack rate represents the proportion of final infected individuals normalized over the community population and serves as an epidemic characteristic. We report these metrics only for datasets with complete infection curves. It is worth mentioning that amongst the datasets, Wi-Fi static networks results have higher difference with its dynamic one. The reason lies in the node activity (number of edges) in the temporal network which is significantly lower in the second half, where we are using for epidemic forecasting. The number of edges per timestamp is presented in Fig. S(4). Overall, our analysis suggests that EdgeMST outperforms DegMST as a proxy for dynamic graphs, and both EdgeMST and DegMST outperform the full static approach. These approaches provide valuable insights for estimating disease characteristics and understanding the potential impact of a pandemic.

#### Observation 2

For epidemic forecasting, the EdgeMST network has the closest maximum fraction of active cases, peak time, and final attack rate to that of the dynamic graph compared to both full static and DegMST.

**Table 3:**
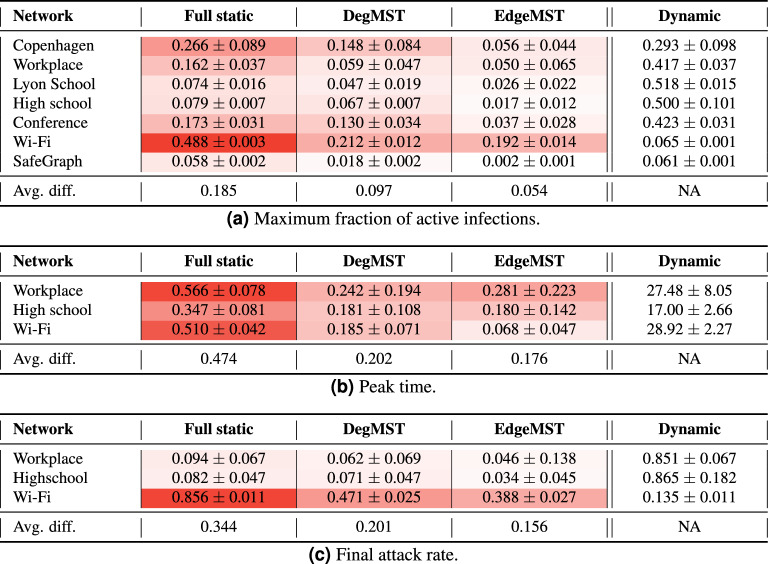
Absolute difference between Full static, DegMST and EdgeMST graphs and the dynamic graph in (a) *maximum fraction of active cases*, (b) *peak time*, and (c) *final attack rate*.

The exact values are reported for the dynamic graph column. We can see that EdgeMST overall has smaller differences across the datasets and metrics (less red), closely followed by DegMST, whereas full static is the farthest (most red).

### Epidemic modelling on aggregated static networks

**Figure 3:**
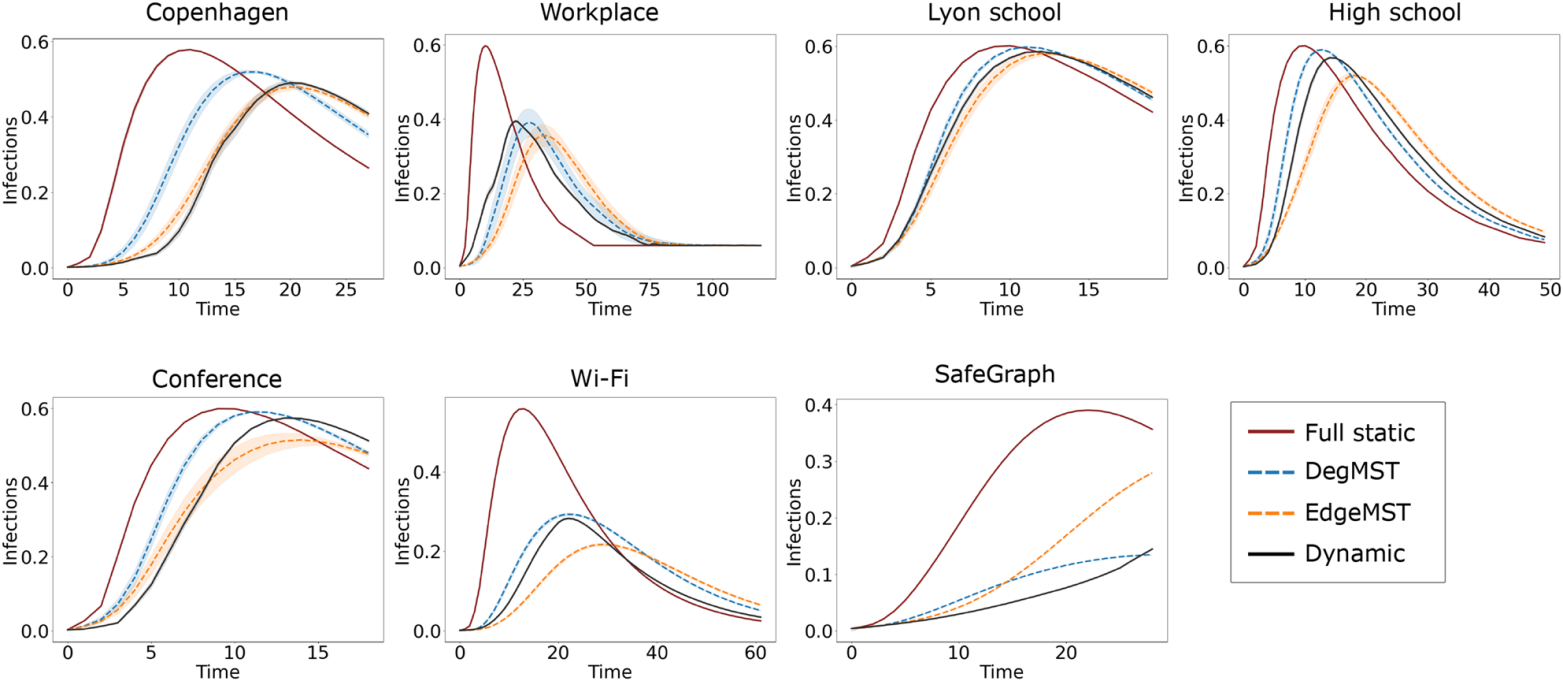
Epidemic modelling on aggregated networks. Unlike Fig. 2, here the static graphs aggregate the full time span, which is not available when forecasting into future. However, the patterns observed are similar to Fig. 2. That is DegMST and EdgeMST have similar active case infection curves to the dynamic graph while the full static graph has major differences, especially on large networks such as Wi-Fi and SafeGraph.

A common approach in approximating static networks from dynamic networks is to aggregate the dynamic graphs over the entire time span using various methods. Researchers then compare the spread of the disease over the static and dynamic networks. In this section, we employ the same approach and present the disease dynamic results using our proposed methods, DegMST and EdgeMST, and compare them with dynamic and full static models. Figure 3 shows the active infection curves of different contact network datasets. In this experiment, the static networks are approximated by aggregating temporal graphs over the full time span. Across all datasets, irrespective of their size, the full static graph tends to overestimate the temporal curves. The active infection curves are more aligned in networks with higher average dynamic edge density, such as Lyon School, High School, and Conference. The higher edge density in these networks are due to a more homogeneous network, which remains active when aggregated into full static or other static graphs. Conversely, and exhibit higher average dynamic edge density, the full static graph demonstrates significant differences from the temporal curve. However, the curves generated by DegMST and EdgeMST closely resemble the dynamic curve. In larger, more realistic networks such as Wi-Fi and SafeGraph, the difference is more significant and the full static graph fails to be a good proxy for the dynamic graph while EdgeMST and DegMST are better proxies. Additionally, we present the cumulative infections in Supplementary Fig. S(3). The KL divergence for Fig. 3 is reported in Supplementary Table S(1) and other disease characteristics are reported in Supplementary Table S(2).

#### Observation 3

For epidemic modelling, EdgeMST and DegMST have closer active infection curves to the dynamic graph based on KL divergence by 15.9% and 15.4% when compared to the full static graph.

### Difference in network structure

The influence of community structure on the spread of infections over networks is a well-recognized phenomenon. In this context, Table 4 presents the global efficiency and algebraic connectivity of various networks. These two global metrics have been shown to play a crucial role in understanding and predicting epidemic spread across different network structures^23^. Global efficiency measures the effectiveness of pathogen or information transmission through a network. In Table 4a, we provide the absolute difference between the global efficiency of three static networks and that of the dynamic network, alongside the precise values for the dynamic graph. Our findings reveal that EdgeMST exhibits the closest global efficiency to the dynamic graph, indicating that it better preserves the overall contact patterns and spreading behavior of the network. On the other hand, both DegMST and the full static approach yield more distant approximations. The second metric, algebraic connectivity, quantifies the level of network connectivity. Table 4b reports the algebraic connectivity of different networks, which is calculated as the second smallest eigenvalue of the Laplacian matrix. A higher value indicates greater network connectivity and faster disease spread. Furthermore, the metric is only positive for connected graphs. In the case of the Wi-Fi and Safegraph datasets, the dynamic graphs are disconnected over time, resulting in an algebraic connectivity value of 0 for these two datasets. EdgeMST and DegMST have closer algebraic connectivity to the dynamic graph compared to full static ones. The same metrics are reported for the graphs used in experiments of Fig. 2 in Supplementary Table S(4). Table 4c reports the maximum node degree over different networks. As seen, full static and DegMST contain high degree nodes which creates hubs for spreading the disease, while maximum node degrees in EdgeMST is closer to dynamic networks.

Our results highlight the significance of using EdgeMST as a reliable proxy for preserving the structures of dynamic networks. By closely aligning with the dynamic graph, EdgeMST allows for a more accurate representation of the underlying contact patterns and spreading dynamics. In contrast, the DegMST and full static approaches exhibit greater deviations from the dynamic network’s global efficiency and algebraic connectivity. Overall, these observations emphasize the importance of considering community structure and employing appropriate static network approximations, such as EdgeMST, to better understand the spreading dynamics of infections within networks.

**Table 4:**
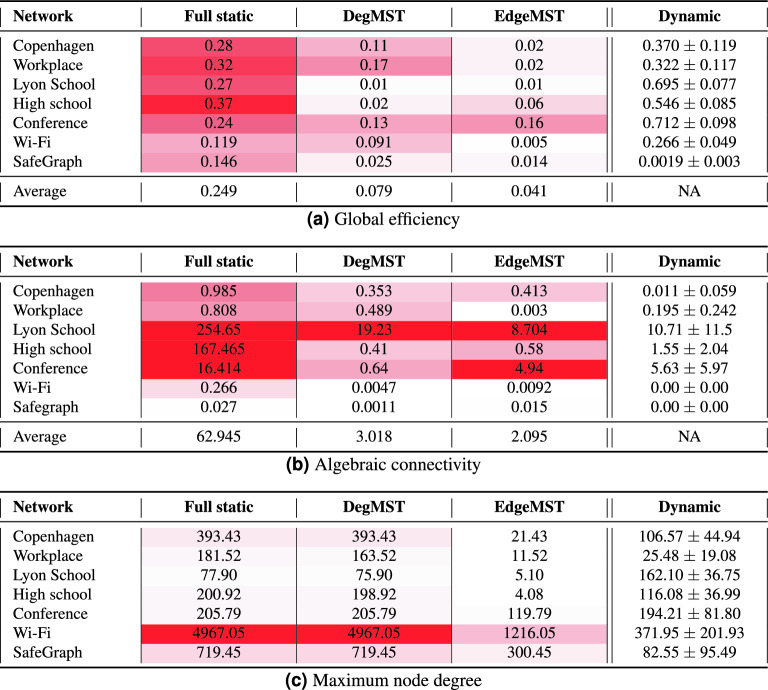
Absolute difference between full static, DegMST and EdgeMST graphs and the dynamic graph in terms of (a) *global efficiency*, (b) *algebraic connectivity*, and (c) *maximum node degree*.

The exact values are reported for the dynamic graph column. We can see that EdgeMST overall has smaller differences across the datasets and metrics (less red), closely followed by DegMST, whereas full static is the farthest (most red).

## Discussion

In this work, we examined the use of past temporal networks to approximate a static network capable of predicting infectious disease spread over a population, while maintaining the dynamic network’s structural characteristics. Our study depicts that the conventional methods used to convert a dynamic network to a static one often leads to severe overestimation of disease characteristics and infection curves. We address this issue by proposing two novel algorithm for converting a dynamic network into a static one, EdgeMST and DegMST. These algorithms are based on the frequency of temporal edges and node degrees, respectively. To evaluate our methods, we compare different epidemic characteristics, such as maximum active infections, peak time and final attack rate, which are important metrics for governments to predict hospitalization loads or placing interventions. Moreover, we measure the closeness of infection curves by KL divergence which showed up to four times closer curves to dynamic ones compared to the full static graph curves. When comparing with dynamic graphs, our methods are capable of being a proxy to be used for epidemic forecasting when the future contact network are not observed yet. Also, when aggregated static networks such as our EdgeMST and DegMST are used, it is easier to preserve the privacy of individuals.

While both of our proposed methods have good performance in epidemic forecasting and modelling, EdgeMST aims to preserve the frequently recurring edges in the temporal graph while DegMST focuses on high degree nodes in the graph. These recurring edges forms the skeleton of the temporal network which are consistent over time (as they appear in most snapshots). Moreover, DegMST focuses on preserving hubs around the high degree nodes, potentially less representative of the temporal network . Therefore, we believe that EdgeMST approach is better at capturing the core structure of the underlying temporal network. One possible future direction is to build upon EdgeMST and DegMST and augment them with additional predicted edges in the future.

## Methods

### Graph notations

We consider a static graph, $\textbf{G} = (\textbf{V}, \textbf{E})$ where $\textbf{V}, \textbf{E}$ are the set of nodes and edges in the graph, respectively. An edge $e = (u,v) \in \textbf{E}$ between nodes *u*,*v* is considered to be undirected, since the contact between two individuals has no inherent directions. Dynamic graphs can be modeled as discrete time and continuous time graphs, from which we use the former in this study. A dynamic contact graph can be represented as a sequence of graph snapshots, $\textbf{G} = \{ \textbf{G}_t \}_{t=1}^{T} = \{ (\textbf{V}_t, \textbf{E}_t) \}_{t=1}^{T}$, where each $\textbf{G}_t = ( \textbf{V}_t, \textbf{E}_t )$ is the graph snapshot at time $t \in [ 1 \dots T ]$. The average node degree across all snapshots is denoted with *k*, which indicates the sparsity. The terms graph and network are used interchangeably in this paper.

### Epidemic modelling

**Classic SEIR** In the classic SEIR model, disease dynamics at each time is calculated as follows^40^:

1 $$\begin{aligned} \frac{dS}{dt} = - \frac{\beta SI}{N}, \; \; \frac{dE}{dt} = \frac{\beta SI}{N} - \sigma E, \; \; \frac{dI}{dt} = \sigma E - \gamma I, \; \; \frac{dR}{dt} = \gamma I \end{aligned}$$

In this model, each individual in the population is assigned to one of the four disease states: *Susceptible* (*S*), *Exposed* (*E*), *Infected* (*I*), and *Recovered* (*R*) at any given time step *t*. Parameters $\beta , \sigma , \gamma$ are the transition rates from *S* to *E*, *E* to *I*, and *I* to *R*, respectively. Reinfections are not considered in this model.

### Contact network SEIR

Classical compartment based models have the homogeneous mixing assumption. At each time step, there is equal chance for individuals to be in contact with each other therefore the spreading patterns of the classical SEIR is equivalent that of a regular random graph with a specified average degree. More specifically, when considering contact networks, the individual transmission from the *S* compartment to the *E* compartment is based on the *transmission probability* $\phi$, derived from $\beta = k \cdot \phi$ where *k* is the average degree of the contact network^41^. After exposure to the disease, individuals will be infected with rate $\sigma$ and then recover with a rate of $\gamma$ same as in standard SEIR model. Algorithm 1 shows how to execute the SEIR model with contact networks. In this algorithm, we give the disease characteristics as well as the dynamic or static network, $\textbf{G}^{\{t\}}$, the total time, T, and the initial set of individuals as inputs. We are considering S/E/I/R to be sets in Algorithm 1, sizes of which corresponds to the quantities in Eq. (1). The number of susceptible individuals at each time step is calculated as: $S = N-|E|-|I|-|R|$ where *N* is the number of nodes. Please note that in a dynamic network the $\textbf{G}^{\{t\}}$ changes at each time step and the disease transmissions are only considered on the active connections in that corresponding timestamp, while in a static network it is the same over time.

**Algorithm 1:**
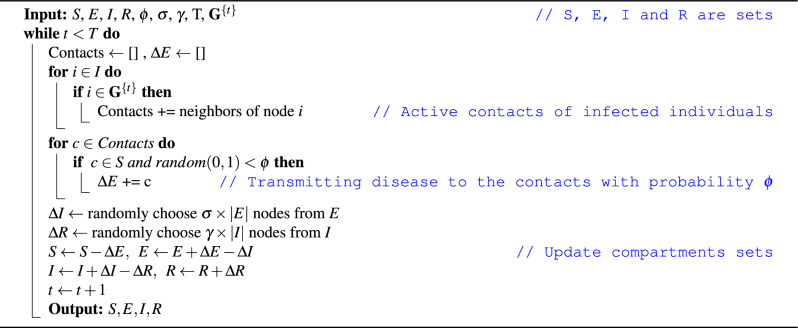
SEIR with contact networks.

### Conversion to static graphs

#### Full static graph

In this section, we discuss algorithms to convert from a dynamic contact graph into a static one. We first present the standard algorithm used in the literature, called the full static graph method. This algorithm collapses all edges in the dynamic graph into a single static graph thus resulting in a full static graph $\textbf{G}_{FS} = (\textbf{V}_{FS}, \textbf{E}_{FS})$ in which $\textbf{V}_{FS}$ and $\textbf{E}_{FS}$ are all nodes and edges existed in the dynamic graph respectively. Therefore, an edge $e \in \textbf{E}_{FS}$ is formed if two individuals was in contact with each other at any time in the dynamic networks. In the case where there are disconnected nodes in the graph, we construct the full static graph by keeping the largest connected component while removing rest of the nodes and their corresponding edges. Similarly, the disconnected nodes and edges are also removed from the dynamic graph. The major limitation of full static graph is that it assumes all edges in the dynamic graph exist together thus losing the sparsity of the dynamic graph.

#### EdgeMST

To preserve the sparsity of the dynamic graph, we propose two novel algorithms to convert a dynamic graph into a static one designed specifically for epidemic modeling purpose. First, we introduce the Edge Minimum Spanning Tree or EdgeMST graph which is constructed to have the same average degree *k* as the dynamic graph, thus preserving the graph sparsity. This is achieved by examining the frequency of each edge in the dynamic graph and adding the edges with highest frequency first to the static EdgeMST graph $\textbf{G}_{EM} = (\textbf{V}_{EM}, \textbf{E}_{EM})$. The algorithm terminates when sufficient edges are added to $\textbf{G}_{EM}$ such that $\textbf{G}_{EM}$ has the same average degree *k* as the dynamic graph $\textbf{G}$. In comparison, the edge frequency information is ignored in the full static graph construction. Another consideration is how to construct static graphs which are connected. This is important because a disconnected graph means some population would never be reachable by the disease thus affecting key characteristics such as maximum number of infections. To guarantee that $\textbf{G}_{EM}$ is connected, we first construct a minimum spanning tree from the full static graph $\textbf{G}_{FS}$ using the Kruskal algorithm^42^. Similar to the full static graph, we retain subgraph containing the largest connected component from the graph when there are disconnected nodes. Both EdgeMST and DegMST algorithms start by generating a Minimum Spanning Tree (MST) from the aforementioned full static graph, which are guaranteed to be a connected graph with one component (as required for finding a MST). Then we add edges based on frequency as discussed above. In this way, we preserves the most frequent edges from the dynamic graph while remaining connected. Algorithm 2 summarize the above.

**Algorithm 2:**
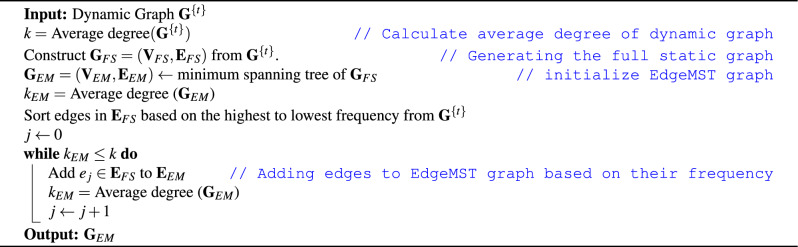
Construct EdgeMST graph.

#### DegMST

Here, we propose an alternative algorithm which preserves the high degree nodes from the dynamic graph, called Degree Minimum Spanning Tree or DegMST. In the DegMST graph $\textbf{G}_{DM} = (\textbf{V}_{DM}, \textbf{E}_{DM})$, we first construct the MST with the Kruskal algorithm, similar to above, then we add edges from the highest degree nodes in the dynamic graph. The node degree is the sum of the degree from all snapshots of a given node. In this way, high degree nodes represents the most consistent individuals who acted as hubs or super-spreaders. This is motivated by recent work which studies the role of super-spreaders in highly infectious disease such as COVID-19 ^43,44^. The DegMST graph $\textbf{G}_{DM}$ is completed when the average degree *k* is the same as the dynamic graph $\textbf{G}$. Full details for the construction of the DegMST graph can be found in Algorithm 3.

**Algorithm 3:**
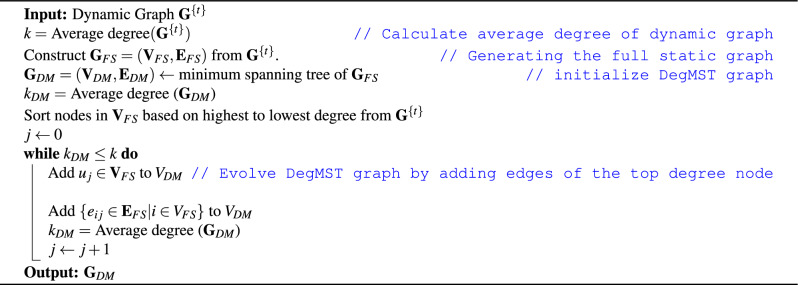
Construct DegMST graph.

### Complexity

For a dynamic graph $\textbf{G}$ with $|\textbf{V}|$ nodes and $|\textbf{E}|$ edges, we discuss the computational complexity of the three conversion algorithms described before. First, for the full static graph $\textbf{G}_{FS}$ algorithm, the complexity is $O(|\textbf{E}|)$ as it simply scans through all edges of the dynamic graph $\textbf{G}$ once. When using $\textbf{G}_{FS}$ in the contact network SEIR model, the time complexity for the disease model would be $O(T \cdot |\textbf{E}_{FS}|)$ where *T* is the number of time steps of the disease model and $|\textbf{E}_{FS}|$ is the number of edges in the full static graph. For our proposed EdgeMST and DegMST graph algorithms, computing the minimum spanning tree has the highest complexity^42^. Therefore, both of them has complexity $O(|\textbf{E}_{FS}| \log |\textbf{V}|)$ where $|\textbf{V}|$ is the number of nodes in the dynamic graph. When running contact graph SEIR with EdgeMST and DegMST, the complexity is $O(T \cdot |\textbf{E}_{EM}|)$ or $O(T \cdot |\textbf{E}_{DM}|)$ respectively. As many edges exist only for a short duration in the dynamic graph, it is possible that $|\textbf{E}_{EM}| \ll |\textbf{E}_{FS}|$ or $|\textbf{E}_{DM}| \ll |\textbf{E}_{FS}|$. Therefore, EdgeMST and DegMST has faster contact graph SEIR run time.

### Supplementary Information

Supplementary Information.

## Data Availability

All data used in this work is publicly available except the SafeGraph, which should be purchased through the company website. The link to other datasets are given here: Copenhagen, Sociopattern datasets(Workplace, Lyon school, high school and Conference) and Wi-Fi.

## Code availability

For reproducibility, the source code is publicly available via Github. Detailed instructions are provided to process datasets and reproduce experiments from this work. We also include example usage and software dependencies. Any inquiries can be directed to the lead authors.
