# Supplementary material for: Static graph approximations of dynamic contact networks for epidemic forecasting
Source: Sci Rep. 2024 May 22;14:11696. doi: 10.1038/s41598-024-62271-0 (PMC11111697; doi:10.1038/s41598-024-62271-0)
Supplement: Supplementary file 1 — Supplementary Information. [file 41598_2024_62271_MOESM1_ESM.pdf]

Supplementary Information

Static Graph Approximations of Dynamic Contact Networks for Epidemic Forecasting

Razieh Shirzadkhani, Shenyang Huang, Abby Leung, Reihaneh Rabbany

Disease dynamics on synthetic networks The SEIR model (or referred to as the standard model), assumes homogeneous mixing among the population. However, in real-world instances, contacts between individuals is dynamic. In Supplementary Figure S(1).a we compare the results of the standard model with those of temporal network, as well as two synthetic networks, regular and random, for Copenhagen dataset. The regular and random networks have an average degree that is the same as the static average degree mentioned in Table 1 and the random graph is regenerated at each time step, so that it is closer to the temporal graph assumption. We also match the transmission probability, ϕ, on graphs and transmission rate, β, in the differential equations based on the average degree of the graph, which is equal to $\phi = 0.135$, as explained in the methodology section. However, since both the transmission probability, ϕ, and transmission rate, β, correspond to the properties of the disease, we fix ϕ for the remaining experiments when comparing different graph models in the simulations. To do this, we fit a regular network to the standard model, which has an average degree of 22, resulting in $\phi = 0.1227$. Using this fixed transmission probability, we still observe that using the regular and random graphs to model the contact network leads to overestimation of the infection curves (Supplementary Figure S(1).b). This is true even when their average degree is matched to the temporal graph, and the graphs are much sparser (Supplementary Figure S(1).c for Copenhagen dataset and Supplementary Figure S(2) for the rest of the datasets).

Furthermore, by comparing Supplementary Figure S(2).a and b we see that the full static graph not only predicts the infection curves way higher than the temporal graph, but also it overshoots the standard SEIR as well. Therefore, neither of these models have acceptable performance on epidemic modelling over a population. In the next section, we present the results of disease spread over our proposed static network approximations and compare them with the full static and dynamic graphs results.

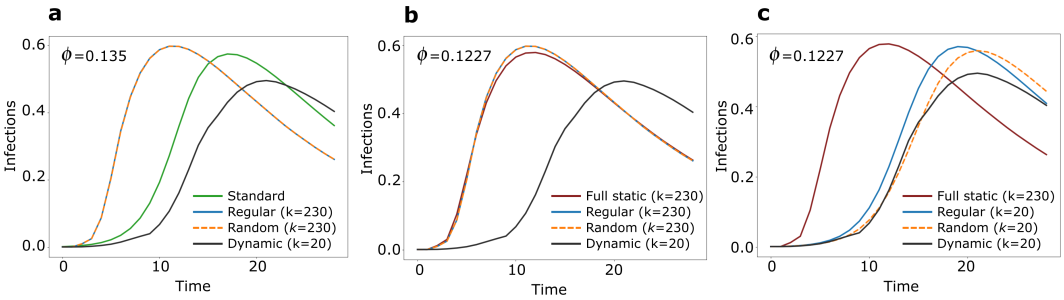

Supplementary Figure S(1). Active infection curves for Copenhagen dataset. a) Standard model overestimates the temporal infection curves. Regular and random graphs overestimate the temporal results even when we use (b) matching transmission probability and (c) matching average degree with temporal network. (Regular and Random completely overlap in (a) and (b))

In Supplementary Figure S(2) the comparison between static and dynamic networks is shown for the rest of the datasets by matching the average degree of the regular and random networks to that of the dynamic network. for the Copenhagen dataset this comparison is shown in Supplementary Figure S(1).c. Although in some networks the regular and random have closer approximation of he dynamic results, in other networks significant differences exist between them. As expected, in datasets with lower temporal edge density, the regular and random networks perform worse compared to datasets with higher temporal average edge density. Therefore, generally, the regular and random graphs are not suitable models for estimating disease spread over a population.

Cumulative infected cases of epidemic modelling The curves of the cumulative infected cases are shown in Supplementary Figure S(3). Generally, the cumulative infected curves of the DegMST and EdgeMST graphs are closest to that of the dynamic network while the full static graph being the most different. Also, as the dataset gets larger, the full static curve gets farther from the dynamic network. In Wi-Fi dataset, the disease does not spread through whole population because some nodes only exist for a brief period thus not reachable by the infection, and interestingly, the DegMST and EdgeMST graphs were able to predict this behavior and the disease dies out before infecting everyone.

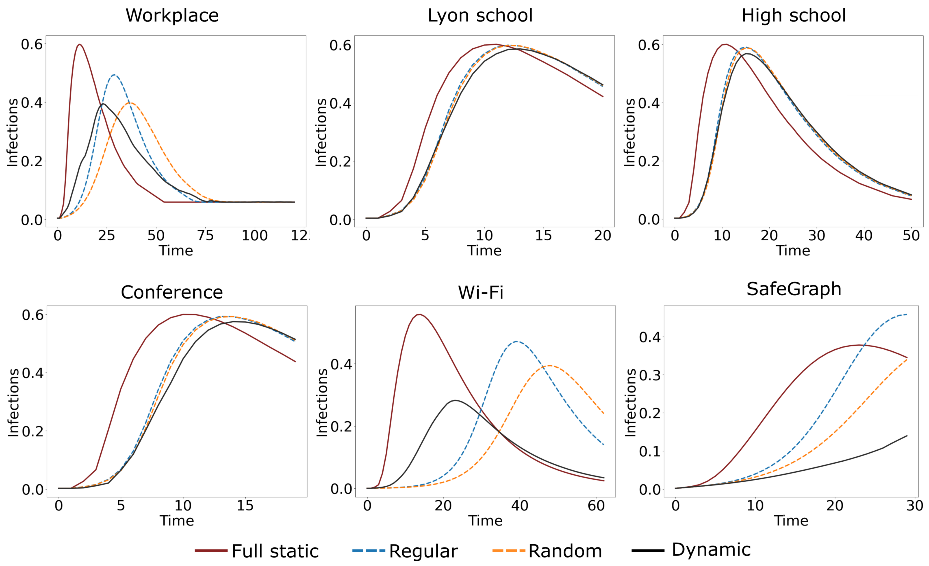

Supplementary Figure S(2). Active infection curves. Contact network structure significantly affects the infection curves. This can be seen when comparing the Full static and temporal results with regular and random graph curves.

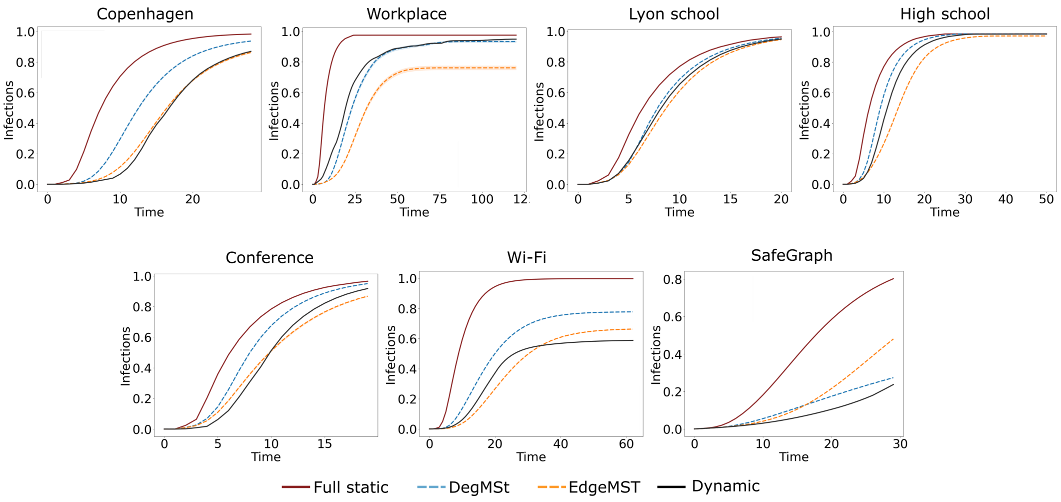

Supplementary Figure S(3). Cumulative infected cases corresponding to Figure 3. DegMST and EdgeMST has similar cumulative infection curve to the dynamic graph while the full static graph has major differences on large networks such as Wi-Fi and SafeGraph (about 300% off).

Supplementary Table S(1). *Kullback–Leibler divergence* between various static networks and the dynamic network active infection curves in Figure 3. EdgeMST and DegMST have much smaller KL divergence than the full static graph.

Network	Full static	DegMST	EdgeMST
Copenhagen	0.571 ± 0.206	0.132 ± 0.110	0.069 ± 0.207
Workplace	0.268 ± 0.069	0.185 ± 0.250	0.192 ± 0.193
Lyon School	0.029 ± 0.017	0.007 ± 0.008	0.010 ± 0.025
High school	0.142 ± 0.048	0.026 ± 0.026	0.032 ± 0.029
Conference	0.142 ± 0.049	0.035 ± 0.024	0.051 ± 0.102
Wi-Fi	0.328 ± 0.039	0.014 ± 0.007	0.086 ± 0.023
SafeGraph	0.054 ± 0.002	0.024 ± 0.002	0.015 ± 0.001
Average	0.219	0.060	0.065

Supplementary Table S(2). Absolute difference between Full static, DegMST and EdgeMST graphs and the dynamic graph in terms of,
a) *maximum fraction of active cases*, b) *peak time*, and c) *final attack rate* corresponding to Figure 3. The exact values are reported for the dynamic graph column. We can see that DegMST overall has smaller differences across the datasets and metrics (less red), closely followed by DegMST, whereas Full static is the farthest (most red).

Network	Full static	DegMST	EdgeMST	Dynamic
Copenhagen	0.082 ± 0.005	0.033 ± 0.011	0.011 ± 0.007	0.496 ± 0.005
Workplace	0.200 ± 0.011	0.086 ± 0.034	0.022 ± 0.017	0.397 ± 0.011
Lyon School	0.015 ± 0.003	0.013 ± 0.004	0.004 ± 0.003	0.586 ± 0.002
High school	0.030 ± 0.005	0.023 ± 0.006	0.037 ± 0.017	0.570 ± 0.005
Conference	0.024 ± 0.003	0.019 ± 0.003	0.039 ± 0.013	0.575 ± 0.003
Wi-Fi	0.276 ± 0.008	0.015 ± 0.009	0.064 ± 0.014	0.282 ± 0.007
SafeGraph	0.246 ± 0.002	0.009 ± 0.002	0.135 ± 0.003	0.144 ± 0.001
Avg. diff.	0.124	0.028	0.044	NA

(a) Maximum fraction of active infections.

Network	Full static	DegMST	EdgeMST	Dynamic
Copenhagen	0.443 ± 0.036	0.190 ± 0.077	0.075 ± 0.063	21.14 ± 1.386
Workplace	0.512 ± 0.024	0.320 ± 0.370	0.497 ± 0.314	22.66 ± 1.124
Lyon School	0.145 ± 0.050	0.052 ± 0.048	0.066 ± 0.075	12.44 ± 0.535
High school	0.313 ± 0.038	0.116 ± 0.066	0.226 ± 0.133	15.18 ± 0.589
Conference	0.294 ± 0.018	0.141 ± 0.049	0.100 ± 0.097	14.16 ± 0.366
Wi-Fi	0.400 ± 0.017	0.045 ± 0.039	0.309 ± 0.074	23.02 ± 0.140
Avg. diff.	0.351	0.144	0.212	NA

(b) Peak time.

Network	Full static	DegMST	EdgeMST	Dynamic
Copenhagen	0.113 ± 0.021	0.069 ± 0.022	0.028 ± 0.026	0.869 ± 0.021
Workplace	0.026 ± 0.006	0.023 ± 0.026	0.042 ± 0.028	0.948 ± 0.006
Lyon School	0.014 ± 0.004	0.007 ± 0.005	0.006 ± 0.010	0.948 ± 0.03
Highschool	0.000 ± 0.001	0.000 ± 0.001	0.012 ± 0.008	0.984 ± 0.001
Conference	0.047 ± 0.006	0.032 ± 0.006	0.050 ± 0.062	0.917 ± 0.006
Wi-Fi	0.409 ± 0.015	0.154 ± 0.022	0.026 ± 0.018	0.587 ± 0.015
Avg. diff.	0.101	0.047	0.027	NA

(c) Final attack rate.

Supplementary Table S(3). Percentage of nodes removed from static and dynamic graphs.

Dataset	Copenhagen	Workplace	Lyon School	High school	Conference	Wi-Fi	SafeGraph
Removed Nodes	0	0	0	0	0	0.1%	3.3%

Supplementary Table S(4). Absolute difference between Full static, DegMST and EdgeMST graphs and the dynamic graph in a) *Global efficiency*, b) *Algebraic connectivity*, and c) *maximum node degree*. corresponding to graphs used in Figure 2. The exact values are reported for the dynamic graph column. We can see that EdgeMST overall has smaller differences across the datasets and metrics (less red), closely followed by DegMST, whereas Full static is the farthest (most red).

**Network**	**Full static**	**DegMST**	**EdgeMST**	**Dynamic**
Copenhagen	0.24	0.11	0.04	0.37 $\pm$ 0.114
Workplace	0.44	0.16	0.04	0.32 $\pm$ 0.112
Lyon School	0.23	0.00	0.01	0.68 $\pm$ 0.084
High school	0.29	0.02	0.07	0.54 $\pm$ 0.107
Conference	0.13	0.16	0.18	0.75 $\pm$ 0.094
Wi-Fi	0.08	0.07	0.12	0.29 $\pm$ 0.029
SafeGraph	0.146	0.025	0.014	0.0019 $\pm$ 0.003
Average	0.222	0.078	0.067	NA

(a) Global efficiency.

**Network**	**Full static**	**DegMST**	**EdgeMST**	**Dynamic**
Copenhagen	0.768	0.140	0.269	0.226 $\pm$ 0.081
Workplace	1.753	0.468	0.151	0.224 $\pm$ 0.277
Lyon School	29.945	7.182	10.172	11.17 $\pm$ 13.91
High school	166.559	0.724	0.768	1.685 $\pm$ 2.396
Conference	3.117	3.884	7.914	8.881 $\pm$ 7.342
Wi-Fi	0.244	$7.8e-4$	0.005	0.00 $\pm$ 0.00
Safegraph	$7.78e-3$	$7.72e-3$	$7.77e-3$	0.00 $\pm$ 0.00
Average	28.91	1.77	2.75	NA

(b) Algebraic connectivity.

**Network**	**Full static**	**DegMST**	**EdgeMST**	**Dynamic**
Copenhagen	286.31	286.31	94.31	106.69 $\pm$ 47.35
Workplace	156.67	126.67	27.67	26.33 $\pm$ 17.00
Lyon School	105.67	53.67	9.67	177.33 $\pm$ 28.52
High school	171.55	171.55	36.45	124.45 $\pm$ 37.45
Conference	228.38	228.38	139.38	165.62 $\pm$ 67.96
Wi-Fi	4405.24	4405.24	1158.24	551.76 $\pm$ 204.04
SafeGraph	74.39	74.39	27.39	32.61 $\pm$ 17.23

(c) Maximum node degree

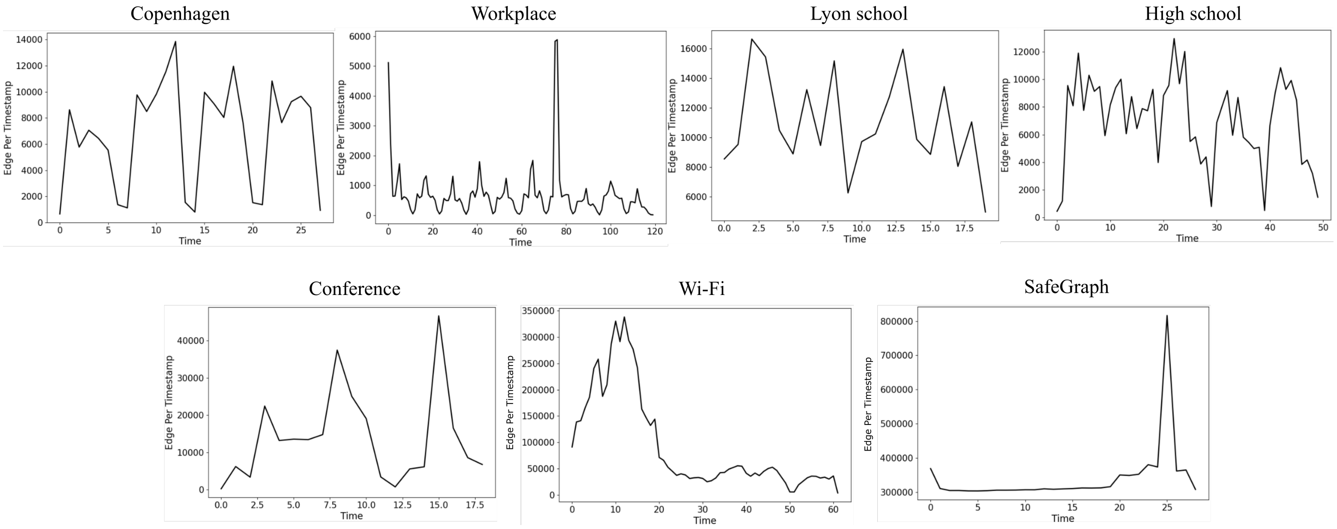

Supplementary Figure S(4). Number of edges per timestamp for different temporal networks.

Comparison with exponential threshold method[9]

We compared our proposed methods with a baseline method from reference[9]. In the exponential-threshold model, a weight is calculated for each edge in temporal network based on the time the edge has occurred, exponentially decaying $e^{-t/\tau}$ with t beign the time of contact and τ is a parameter value. Then all the weights for one identical edge are summed and if the weight is higher than a specific parameter Ω the edge is added to the exponential threshold static network. For each dataset, it is needed to find the best τ and Ω. Here, we use the optimal parameters reported in the paper for *Conference* dataset. We scaled τ parameter to match our time granularity ($\tau = 0.336$ and $\Omega = 0.020$) to generate the exponential threshold static network.

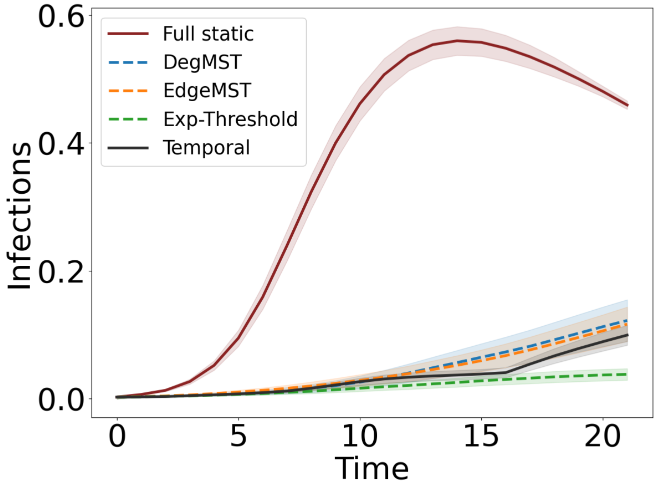

Supplementary Figure S(5). Number of active infections in Conference dataset for different networks. The Exp-threshold network is calculated by following the method in reference[9]. We use the parameters $\tau = 0.336$ and $\Omega = 0.020$.

It is worth mentioning that there are two versions of the Conference data and for more direct comparison, we use the version same as the prior work though it differs from the version we presented in the submission. The key difference is in the edge counts, although the number of nodes remains consistent between the two version. The version that we have used in the paper, considers a contact between two individuals (nodes) if they have been present in a conference area at the same time while the version we are reporting here limits the contacts to those who have less than 1.5m distance from each other.

The results shown in Figure S(5) and Table S(5) are based on epidemic modelling. We have used the same disease constants in all the experiments. The results shown are averaged through 50 runs and normalized by the total number of nodes in temporal network. The exponential threshold method does not retain the number of nodes in the temporal network. For example, the conference temporal dataset contains 403 nodes, while the exponential threshold method only retains 100 nodes. In our proposed methods, we keep all the nodes from temporal networks from the static ones as we construct a minimum spanning tree first.

Supplementary Table S(5). Kullback–Leibler divergence between static networks and the dynamic network active infection curves presented in Figure S(5)

Network	Full static	DegMST	EdgeMST	Exp-threshold
Conference	0.213± 0.041	0.206± 0.204	0.134± 0.182	0.191± 0.223
